# Computational Genomics in the Era of Precision Medicine: Applications to Variant Analysis and Gene Therapy

**DOI:** 10.3390/jpm12020175

**Published:** 2022-01-27

**Authors:** Yung-Chun Wang, Yuchang Wu, Julie Choi, Garrett Allington, Shujuan Zhao, Mariam Khanfar, Kuangying Yang, Po-Ying Fu, Max Wrubel, Xiaobing Yu, Kedous Y. Mekbib, Jack Ocken, Hannah Smith, John Shohfi, Kristopher T. Kahle, Qiongshi Lu, Sheng Chih Jin

**Affiliations:** 1 Department of Genetics, School of Medicine, Washington University, St. Louis, MO 63110, USA; yung-chun@wustl.edu (Y.-C.W.); c.m.choi@wustl.edu (J.C.); shujuanzhao@wustl.edu (S.Z.); k.mariam@wustl.edu (M.K.); kuangyingyang@wustl.edu (K.Y.); fup@wustl.edu (P.-Y.F.); w.max@wustl.edu (M.W.); xiaobing@wustl.edu (X.Y.); 2 Department of Biostatistics & Medical Informatics, University of Wisconsin-Madison, Madison, WI 53706, USA; ywu423@wisc.edu; 3 Department of Pathology, Yale School of Medicine, New Haven, CT 06510, USA; garrett.allington@yale.edu; 4 Department of Neurosurgery, Massachusetts General Hospital, Boston, MA 02114, USA; HSMITH20@mgh.harvard.edu (H.S.); kahle.kristopher@mgh.harvard.edu (K.T.K.); 5 Department of Computer Science & Engineering, Washington University, St. Louis, MO 63130, USA; 6 Department of Neurosurgery, Yale University School of Medicine, New Haven, CT 06510, USA; kedous.mekbib@yale.edu (K.Y.M.); jack.ocken@yale.edu (J.O.); john.shohfi@yale.edu (J.S.); 7 Division of Genetics and Genomics, Boston Children’s Hospital, Boston, MA 02115, USA; 8 Departments of Pediatrics and Neurology, Harvard Medical School, Boston, MA 02115, USA; 9 Broad Institute of MIT and Harvard, Cambridge, MA 02142, USA; 10 Department of Pediatrics, School of Medicine, Washington University, St. Louis, MO 63110, USA

**Keywords:** rare variant, common variant, statistical genetics, genomics, bioinformatics, gene therapy, precision medicine

## Abstract

Rapid methodological advances in statistical and computational genomics have enabled researchers to better identify and interpret both rare and common variants responsible for complex human diseases. As we continue to see an expansion of these advances in the field, it is now imperative for researchers to understand the resources and methodologies available for various data types and study designs. In this review, we provide an overview of recent methods for identifying rare and common variants and understanding their roles in disease etiology. Additionally, we discuss the strategy, challenge, and promise of gene therapy. As computational and statistical approaches continue to improve, we will have an opportunity to translate human genetic findings into personalized health care.

## 1. Introduction

Over the past decade, genome sequencing technology has been one of the fastest growing fields in biomedical science. Thanks to the progress in sequencing automation, the cost of sequencing has dropped dramatically. As a result, an enormous amount of genomic data has been generated, providing an informative profiling of human genetic variations, disease-related mutations, and association between genotype and phenotype [1,2,3,4].

With the achievement of the Human Genome Project and the HapMap Project in the early 2000s, human genetic research in complex diseases started a new chapter: genome-wide association studies (GWAS). In 2005, a landmark GWAS found two single nucleotide polymorphisms (SNPs) associated with age-related macular degeneration [5]. Later, GWAS identified many risk loci associated with diseases and traits, including coronary heart disease [6], obesity [7,8], type 2 diabetes [9], schizophrenia [10], and so forth. As of 11 November 2021, the NHGRI-EBI GWAS catalog has documented 5457 publications and 318,587 associations [11]. Although these associations have led to novel insights into the genetic architecture underlying numerous complex traits, individual common variants tend to have weak effect sizes, and all common variants only explain a moderate proportion of heritability [12]. This lingering gap of “missing heritability” suggests that rare variants (defined as those genetic variants with a population allele frequency less than 1%) that are difficult to detect by GWAS, and possibly the interplay between common and rare variants, may play a major role in complex disease etiology.

**Figure 1:**
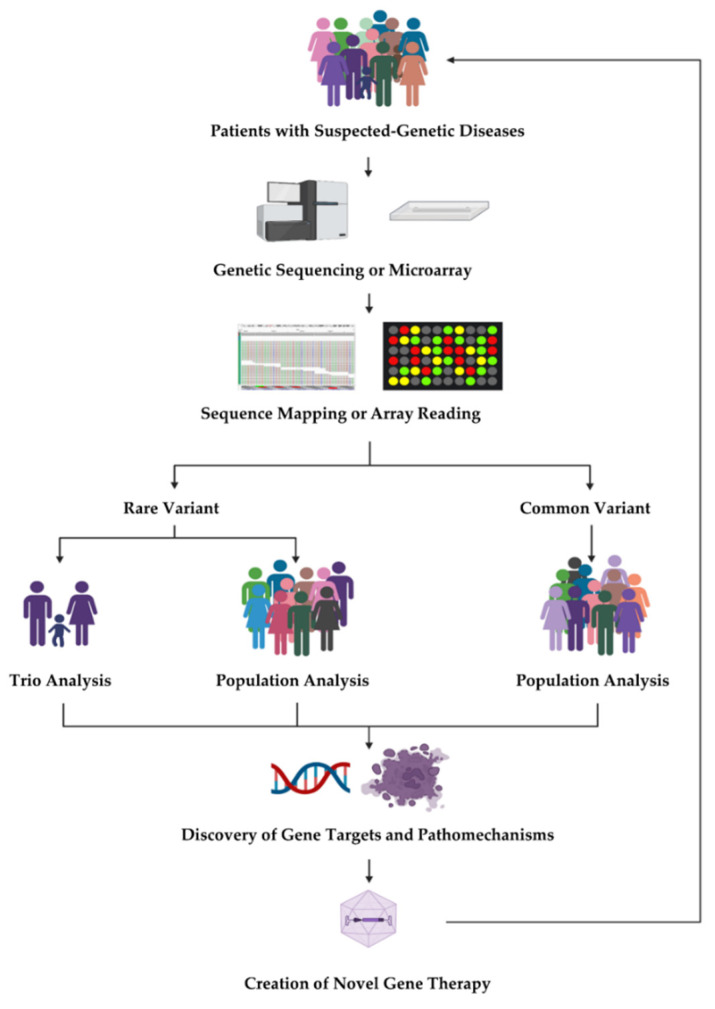
Overview of base pairs-to-bedside approach. Advances in genomic analysis, precision medicine, and gene therapy allow for the genetic evaluation of sporadic and inherited variants in families and large cohorts. Further elucidation of genetic etiology and disease pathomechanisms through genomic and integrative multi-omics studies then catalyze the production of new therapeutic options such as gene therapy for patient care.

With rapid advances in DNA sequencing technologies, assessment of rare genetic variants in complex traits has become feasible. In particular, whole-exome sequencing (WES) and whole-genome sequencing (WGS) have gained popularity in recent studies on gene discovery. Herein, we review the recent analytical approaches for identifying disease-associated rare variants in population-based or family-based studies based on WES or WGS. We also discuss recent advances in common variant association analysis and polygenic risk score methods. Finally, we discuss how to translate genetic discovery into effective therapeutics or treatments. The flow diagram is illustrated in Figure 1.

## 2. Rare Variant Analysis in Unrelated Individuals

A major challenge in rare variant analyses for complex traits is the limited statistical power to identify individual variant associations due to the low allele counts. For example, given a balanced case-control study of 3 K subjects (1.5 K cases vs. 1.5 K controls) at a type I error α of 5 × 10^−8^ and a relative risk of 3, the power to detect a variant with minor allele frequency (MAF) equal to 0.5% is around 0.05. To boost statistical power, most rare-variant association methods combine association signals across multiple rare variants in pre-defined variant sets (e.g., genes, genomic regions, pathways, and functional annotations) and generally assume the presence of multiple trait-associated variants in the same variant set [13]. We note several popular methods below.

The combined multivariate and collapsing (CMC) test is one of the first methods to empower rare variant association analysis by collapsing all rare variants into a single test [14]. A later study introduced the variable threshold (VT) method, which improves statistical power by dynamically selecting the optimal MAF cutoff that distinguishes causal rare variants from nonfunctional variants with higher allele frequencies [15]. The development of the sequence kernel association test (SKAT) is particularly important because it allows for the incorporation of covariates and can also consider rare variants with opposite effect directions [16]. Other methods for studying the rare variant associations, including the cohort allelic sums test (CAST) [17], weighted sum test (WST) [18], the kernel-based adaptive clustering method (KBAC) [19], the versatile gene-based association study (VEGAS) [20], the gene-based association test that uses extended Simes procedure (GATES) [21], the multivariate association analysis using score statistics (MAAUSS) [22], and multi-trait analysis of rare-variant associations (MTAR) [23], have since been developed with subtle nuance in their algorithms. A summary of these methods is shown in Table 1. We also note that study designs, inference algorithms, and statistical details of many approaches have been extensively reviewed by Lee et al. [24].

**Table 1:** Statistical approaches for population-based or family-based rare variant analyses.

Type	Methods	Strengths	Weaknesses	Ref.
Rare variant analysis in unrelated individuals	Combined Multivariate and Collapsing (CMC) test	- More powerful and robust for analyzing a set of rare variants than testing each variant individually	- Reduced power when the grouped variants have effects in opposite directions	[14]
	Variable Threshold (VT)	- Makes no assumption about the causal variant’s allele frequency - Boosts power using functional annotations that give higher weights to functional variants	- Reduced power when the set of variants grouped together have effects in opposite directions - High computational burden for permutation test	[15]
	Sequence kernel association test (SKAT)	- Considers rare variants with opposite effect directions - Test statistics have a closed form approximation for their null distribution - Computationally efficient - Can adjust for covariates	- Less powerful when causal variants have the same effect direction	[16]
	Cohort allelic sums test (CAST)	- More powerful and robust for analyzing a set of rare variants than testing each variant individually	- Reduced power when the grouped variants have effects in opposite directions	[17]
	Weighted sum test (WST)	- Can account for linkage disequilibrium (LD) between variants	- Lower statistical power given few causal variants within a gene	[18]
	Kernel-based adaptive clustering method (KBAC)	- Has higher statistical power in the presence of variant interaction	- No closed form null distribution for test statistics - High computational burden	[19]
	Versatile gene-based association study (VEGAS)	- Only uses summary statistics as input - Can account for LD between variants	- Less powerful for detecting a large gene with many typed non- causal variants - High computational burden	[20]
	Gene-based association test that uses extended Simes procedure (GATES)	- Only uses summary statistics as input - Can account for LD between variants - Variants can have opposite effect directions - Computationally efficient	- Designed for genome-wide association studies (GWAS) and has lower power in rare variant analysis	[21]
	Multivariate Association Analysis using Score Statistics (MAAUSS)	- Leverages multiple phenotypes to improve statistical power	- High computational burden	[22]
	Multi-trait analysis of rare-variant associations (MTAR)	- Improved statistical power in multi-trait multi-variant association analysis - Only uses summary statistics as input	- Relies on a concordant common and rare variant genetic correlation between traits	[23]
De novo variants analysis	DeNovoWEST	- Estimates positive predictive values of each DNV being pathogenic - Incorporates a gene-based weighting strategy	- Limited to exome	[4]
	Chimpanzee–human divergence model	- Estimates the relative locus-specific rates of DNVs	- Can only be applied to a selected candidate gene set	[25]
	denovolyzeR	- Adjusts for sequence depth and the divergences based on human–chimp differences - Does not require any control samples for comparison	- Relies on a pre-computed tabulation of the probability of DNVs arising in each gene - Limited to exome	[26]
Autosomal recessive variant analysis	Resampling-based statistical framework	- Leverages trio data to compare the observed number of recessive genotypes with the empirically estimated counts under the null - Accounts for confounding due to population stratification and consanguinity	- Limited to exome - Strong assumption that all subjects’ genotypes are independent	[27]
	Sampling the observed genotypes and phenotypes by chance	- Incorporates the probabilities of sampling the observed genotypes and phenotypes by chance - Incorporates the phenotypic similarity of patients with the same recessive candidate gene - Corrects for gene-specific levels of autozygosity - Takes account of population structure	- Limited to exome - Requires systematic genotype and phenotype data on a known number of families - Difficult to perform when recording of phenotype terms is incomplete and inconsistent	[28]
	The phased haplotypes-based framework	- Uses the phased haplotypes from unaffected parents to estimate the expected number of biallelic genotypes in affected probands - Accounts for the fact that some fraction of the variants expected by chance are actually causal	- Limited to exome - Strong assumption that all subjects’ genotypes are independent - Strong assumption of full penetrance of all genotypes	[29]
Joint analysis of transmitted variants and DNVs	Transmission and de novo association test (TADA), extTADA	- TADA is the first method developed to jointly model de novo and transmitted mutations by a hierarchical Bayesian modeling framework - extTADA performs a Markov chain Monte Carlo for the Bayesian analysis	- Both are limited to exome - Both cannot incorporate recessive genotypes and model across disease traits	[30,31]
	TADA-Annotations (TADA-A)	- Can combine information on all DNVs in both coding and nearby non-coding regions across studies	- Cannot incorporate transmitted variants	[32]
	TADA-Recessive (TADA-R)	- Can integrate signals from DNVs, transmitted dominant, and transmitted recessive variants	- Limited to exome	[33]
	Multi-trait TADA (M-TADA)	- Can jointly analyze DNVs from multiple traits	- Limited to exome - Cannot incorporate transmitted variants - Can only perform pair-wise comparison	[34]
X-linked variant analysis	Various XCI modes integrated statistical approach	- Considers all X-linked processes (random, skewed, and escaped XCI) - Performs a permutation-based procedure to assess the significance with well-controlled type I error rate	- Has lower power in the random or escaped XCI test - Cannot provide accurate effect size estimate in the escaped XCI model	[35]
	1 and 2 degree-of-freedom tests for association	- Easy to implement using the contingency table approach	- False assumption of equal phenotypic effects between males’ hemizygotes and females’ homozygotes - Does not consider nonrandom XCI and escape from XCI	[36]
	Distinct XCI processes combined using a modified Fisher’s method	- Considers all X-linked processes (random, skewed, and escaped XCI) - Is the most statistically efficient and not sensitive to the unknown biological models	- Strong assumption that all subjects’ genotypes are independent - Cannot adjust for covariates	[37]
	Sex-specific burden analyses	- Can estimate the fraction of probands attributable to rare X-linked variants	- Strong assumption of a monogenic model with full penetrance - Wide confidence intervals for several key parameters	[38]
Digenic variant analysis	The genetic linkage method	- Takes account of phenocopies and reduced penetrance - Able to deal with allelic heterogeneity - Able to identify rare alleles that are present in small numbers of families	- Requires pedigrees of related individuals (and parents’ samples) - Not suitable for common or complex-trait diseases - Unable to deal with high dimensional data and non-linear regression tests	[39]
	The candidate gene approach	- Useful as the first step in exploring known pathways in complex diseases - Offers high statistical power and is computationally efficient	- Subjective in the process of choosing specific candidate genes - Lack of replication studies - Relies on prior hypotheses about disease mechanisms - Unable to deal with high dimensional data and non-linear regression tests	[40]
	Case-only study design	- No need for control recruitment - Improved statistical power compared to the case–control design - Less multiple-testing correction	- Potential increase in type I error rate if the independence assumption is violated - Unable to deal with high dimensional data and non-linear regression tests	[41]
	Random forests	- Broad applications in data mining and machine learning - Flexible and powerful statistical learning tools for analysis - Relatively fast and can handle big GWAS	- Sensitive to insufficient training data, confounding effects, reproducibility, and accessibility - Potential slow-performing algorithm when dealing with large data set - Requires much computational power and resources	[42]

Association analysis methods are ordered and grouped by different types of genetic variants. Each method for certain types of genetic variants is listed in middle column. The references are indicated in the last column.

## 3. Rare Variant Analysis for Family-Based Studies

Family-based association analysis has become increasingly popular in sequencing studies because it provides an opportunity to identify genetic variants that complement the findings in studies of unrelated individuals. The ability to determine whether genetic variants segregate with disease status within families helps distinguish causal variants from non-causal variants [43]. The trio-based study design makes it possible to distinguish between de novo variants (DNVs) and transmitted variants [44,45]. Finally, family-based designs can employ both between- and within-family comparisons in a two-step analysis to increase statistical power while staying robust to population stratification and other confounding factors [46,47,48,49].

### 3.1. De Novo Variant

Spontaneously arising DNVs—those present in proband but absent in parents—play an important role in the pathogenesis of rare congenital diseases such as congenital heart disease [27,45,50,51]. On average, every subject carries one DNV affecting the protein-coding region of the genome [52,53]. However, modeling DNVs has proven to be challenging because DNVs are not distributed equally across the genome and the sequencing depth and distribution vary across sequencing platforms when combining samples from different cohorts.

Several nuanced approaches have been developed to address these issues (Table 1). The O’Roak study was the first to estimate the relative locus-specific rates of DNV by incorporating locus-specific transition, transversion, and indel rates, gene length, and a null expectation based on chimpanzee–human genome differences. However, one major limitation of this approach is that it can only be applied to a selected candidate gene set [25].

To overcome this limitation and more broadly estimate the mutation rates, Samocha et al. developed a de novo expectation model to quantify the mutation rates based on trinucleotide sequence contexts and functional annotations, while adjusting for sequence depth and the divergences based on human–chimp differences [54]. Importantly, this method does not require any control samples for comparison, but instead quantifies the enrichment of synonymous DNVs as a negative control group. Furthermore, this Poisson testing framework for DNV enrichment can yield high statistical power that is difficult to achieve in case–control analysis. An R package called “denovolyzeR” was developed to implement this statistical framework [26].

More recently, Kaplanis et al. developed a method named DeNovoWEST to detect gene-specific enrichments of damaging DNVs. DeNovoWEST is a simulation-based approach that scores all classes of variants on a unified, empirically estimated severity scale quantifying pathogenicity [4]. Compared with denovolyzeR, DeNovoWEST incorporates a gene-based weighting strategy derived from the deficit of protein truncating variants in the general population (e.g., pLI scores) [55]. In the future, incorporation of functional genomic information (e.g., gene expression in disease-relevant tissues) and other variant prioritization metrics may further improve the performance of risk gene identification.

### 3.2. Autosomal Recessive Variant Analysis

To analyze recessive variants that include both homozygous and compound heterozygous variants, a case–control burden test can be performed. However, the challenge in case–control analysis lies in the often distinct ethnic composition and variable degrees of consanguinity (i.e., marriage between closely related relatives) across study cohorts or between cases and controls. Further, it is difficult to establish genome-wide significant associations in case–control comparisons when studying ultra-rare recessive genotypes due to limited statistical power [27].

Several analytical strategies have been developed to address these issues (Table 1). Nadia et al. developed a statistical approach that incorporated the probabilities of sampling the observed genotypes and phenotypes by chance and applied it to a cohort of 4125 families with rare and genetically heterogeneous developmental disorders to identify four novel autosomal recessive disorders [28]. Another study, by Jin et al., developed a resampling-based statistical framework that leverages trio data to compare the observed number of recessive genotypes with the empirically estimated counts under the null. This approach enables a powerful enrichment test while accounting for confounding due to population stratification and consanguinity [27]. Using this approach, they found recessive variants are enriched in distinct biological pathways separate from those implicated by other forms of inheritance and demonstrated that consanguinity is a stronger driver of the recessive form of birth defects [27].

More recently, Martin et al. devised a new approach to use the phased haplotypes from unaffected parents to estimate the expected number of biallelic genotypes in affected probands. Despite methodological differences in these approaches, recent studies unequivocally suggested that recessive coding variants only account for a small proportion of patients with rare congenital disorders (in the range of 1–4%), compared with 10–20% explained by coding DNVs [27,28,29]. The large proportion of unexplained patients even amongst those with affected siblings or high consanguinity suggests that complex inheritance (e.g., oligogenic and polygenic inheritance, gene–environment interaction) or other genetic variations (e.g., non-coding regulatory elements or structural variants) await discoveries using improved genomic technologies and statistical methods in the future.

### 3.3. Joint Analysis of Transmitted Variants and DNVs

Recent sequencing-based studies have revealed that disease risk genes could be affected by multiple types of genetic variations (e.g., DNVs, transmitted rare variants, or regulatory variants) [27,44,56]. To accelerate risk gene discovery, several groups have developed a novel statistical framework, known as the Transmission and De novo Association (TADA) test, to combine information from multiple types of genetic variations or across multiple genetically correlated disease phenotypes (Table 1). While these tools have been proven effective, there are some differences and limitations of each TADA variation. We provide a brief overview below.

The original TADA approach and an extended approach, extTADA, were designed to incorporate DNVs and transmitted dominant variants in proband-parent trios, as well as variants identified in unrelated cases and controls for risk gene mapping. A hierarchical Bayesian strategy is used to rank and test risk genes for a disease of interest [30,31]. However, these approaches fail to consider variants in the non-coding genome. Liu et al. employed an approach called TADA-Annotations (TADA-A), which combines information of all DNVs of a gene in both coding and nearby non-coding regions to maximize the power to detect risk genes [32]. The authors applied TADA-A to WGS data of ~300 ASD family trios and found that the contribution of de novo non-coding mutations could be comparable to that of de novo loss-of-function or missense mutations in the coding regions, which suggests that incorporation of non-coding variants from WGS data can aid risk gene discovery.

Another limitation of the original TADA approach is that it does not consider the contribution from recessive variants. This limitation has been addressed by TADA-Recessive (TADA-R), which is built upon TADA to include DNVs, autosomal dominant variants, and autosomal recessive variants [33]. By applying TADA-R to 2645 congenital heart disease-affected family trios, Li et al. identified 15 significant genes, half of which are novel, leading to new insights into the genetic basis of congenital heart disease and once again highlighting the importance of including recessive variants in genetic studies [33].

The development of multi-trait TADA (mTADA) coincided with the need for the ability to perform a joint analysis of DNVs from multiple genetically correlated disease traits to increase the statistical power for risk gene discovery [34]. The mTADA approach uses the expectation–maximization algorithm to draw associations between the two diseases. By applying mTADA to large datasets consisting of more than 13,000 trios for five correlated neuropsychiatric disorders and congenital heart disease, the authors reported additional risk genes and provided new insights into the shared and disorder-specific biological mechanisms across these disorders [34].

## 4. X-Linked Variant Analysis

The sex chromosome constitution is one major source of genetic variation in humans [57]. Moreover, there are many differences in the phenotypes between females, who typically have two X chromosomes, and males, who typically have one X and one Y chromosome. However, the impact of genetic variations on the sex chromosomes has been largely overlooked in genetic association studies. Additionally, the complex and dynamic X chromosome inactivation (XCI) creates challenges in X-linked variant analyses [35,58]. XCI, as first described by Ohno et al. in 1959, usually occurs randomly for one of the two X chromosomes in females to equalize dosage of gene products from the X chromosomes between males and females [59]. Conventional approaches for X-linked variant analysis, such as the Cochran–Armitage test, assume equal phenotypic effects between males’ hemizygotes and females’ homozygotes (Table 1) [36]. However, recent studies showed that genes on the silenced X chromosome can be nonrandomly selected for inactivation and some can escape from XCI [35,60,61]. Thus, the contingency table approach could lead to a significant power loss if the underlying biological mechanisms are nonrandom or escaped XCI.

To address this, Wang et al. took various XCI modes (i.e., random, nonrandom, or escaped XCI) into consideration, and proposed a new statistical approach with greater statistical power in which 0 or 2 were used for genotype coding in males and 0, d, or 2 were used in females. Here, d quantifies females’ heterogeneous effective allele counts (Table 1) [35]. Although the improved efficiency and robustness of this approach are suitable for genome-wide analysis, this method did not consider linkage disequilibrium (LD) and lacked the ability to adjust for covariates such as age, which is likely to affect the XCI ratio [37,62,63].

The recent development of very large WES cohorts such as the Deciphering Develop-mental Disorders project, coupled with the improved understanding of the germline mutation rate, have enabled more robust estimation of the absolute and relative fraction of inherited variants and DNVs for complex diseases. Martin et al. conducted sex-specific burden analyses of damaging DNVs to identify an enrichment of specific classes of X-linked variants in probands and estimated the fraction of probands attributable to those variants [38]. They found that such variants do not fully account for the differential prevalence between the sexes and that the bulk of X-linked burden is in known developmental disorder-associated genes [38]. More robust X-linked variant analysis and better under-standing of sex differences in X chromosome biology will require even larger cohorts and integration of multi-omics data (e.g., RNA-seq or ATAC-seq) that can suggest which X chromosome is silenced and to what degree a gene is expressed on the inactivated X chromosome.

## 5. Digenic Variant Analysis

Digenic inheritance (DI) refers to the simplest form of oligogenic inheritance [64]. Individuals with digenic diseases harbor two risk variants at two genomic loci that correspond to the development of phenotypes that do not segregate in the typical Mendelian inheritance fashion. While thousands of variants have been discovered and linked to monogenic diseases, only a few hundred were linked to 54 digenic disorders according to the DIDA database (http://dida.ibsquare.be/, accessed on 17 November 2021). This can be attributed to several factors, including difficulties in establishing a genotype–phenotype correlation, reduced penetrance, phenotypic and expression variability, and most importantly, the lack of efficient and robust methods for detecting gene–gene interaction due to the overall small effect of each variant on disease risk. The genetic linkage analysis method was successful in detecting digenic diseases in some families [39], but other methods can be used specially when the parents’ samples are not available for segregation analysis (Table 1). For example, the candidate gene approach was very useful in some cases where a gene of interest is selected to be investigated based on its relevance to the pathway(s) involved in the development of the disease [40]. The approach is quick, cheap, and offers high statistical power. However, it has been faced with criticisms due to the lack of replication studies and how much is known about the biological aspect of the investigated disease [65]. Nowadays, the case-only and machine learning approaches are heavily and continuously developed for the prediction of digenic diseases.

### 5.1. Case-Only Approach

The case-only design provides an estimation of gene–gene interactions without requiring negative control samples [66] and demonstrates improved statistical power compared to the case–control design [67,68]. Recently, Kerner et al. proposed a genome-wide, case-only study based on WES data [41]. This approach uses each gene as the unit of analysis and tests all pairs of genes to detect gene-pair interactions underlying diseases. Furthermore, Kerner et al. used a classic variant aggregation approach to combine multiple variants within a gene, and the CAST approach was used to perform burden tests, allowing for further improved statistical power. The proposed method appears to be simple and flexible to apply, with a major advantage of the eliminated need for control recruitment. Moreover, performing hypothesis testing at the gene level greatly reduces the burden of multiple testing and computational time. However, this approach is not robust to gene–gene correlation (e.g., variants in LD) and will have substantially inflated type I error if the independence assumption is violated.

### 5.2. Machine Learning

Although the aforementioned methods have contributed significantly to unraveling oligogenic diseases, they are often met with limitations and criticism, predominantly due to their inability to deal with high dimensional data and non-linear regression tests. For these reasons, machine learning methods started to gain recognition and popularity in the field of genetics, particularly supervised machine learning where the algorithm predicts potential gene–gene interaction as an output depending on the input data and the set of rules obtained through model training. Among the supervised machine learning models, random forests (RFs), neural networks, cellular automata, and multifactor dimensionality reduction are the most used [69]. RFs, a tree-based ensemble approach with several decision-tree classifiers, is especially popular in the field. Where each tree in the forest is trained with a set of data to predict the outcome, in this context the RFs algorithm would predict the gene–gene interaction causing the phenotype in question [42]. The Oligogenic Resource for Variant AnaLysis (ORVAL), which has been used to study digenic diseases, is also a popular online platform that integrates innovative machine learning methods for combinatorial variant pathogenicity prediction with visualization techniques [70,71,72,73]. The candidate digenic predictions are then used to rank gene pairs and build an interactive oligogenic network that can be further explored.

It is understandable that traditional methods alone are unable to detect digenic variants due to the limitations imposed by the used statistical tests and the often-required pre-knowledge of biological aspects of diseases. Likewise, limitations can be faced with the machine learning approach due to insufficient training data, confounding effects, reproducibility and accessibility, and the potential slow-performing algorithm when dealing with large data sets [74,75]. Furthermore, the lack of large case–control cohorts hinders the chances of conforming causative genetic variant combinations. Recent studies on oligogenic diseases provide evidence of the crucial need to combine genetic analysis methods along with functional and experimental studies for validation. Li et al. have provided the first experimental evidence of oligogenic inheritance in heterotaxy, using sequencing analysis and functional studies on zebrafish and mouse [76]. Additionally, Gifford et al. published interesting findings of a family with affected children suffering left ventricular non-compaction cardiomyopathy (LVNC) [77]. In their study, affected children were found to harbor three genetic variants that were proven to cause LVNC when combined all together. CRISPR-Cas9 technology and human induced pluripotent stem cells were used for validation. This suggests that traditional methods alone are not efficient to detect or confirm the subtle effect of combined genetic variants, and that the use of advanced gene-editing coupled with in vivo/in vitro approaches is necessary in future diagnosis of oligogenic diseases.

## 6. Common Variant Association Analysis

A GWAS aims to identify associations between (typically millions of) SNPs and a disease or trait of interest. SNP genotypes are usually obtained using a genotyping microarray for a set of pre-determined variants. The genotype information for each bi-allelic SNP is stored as the count of a reference allele, which can be coded as 0, 1, or 2. It is also a common practice to impute relatively common but ungenotyped SNPs based on a population haplotype reference panel [78]. A GWAS performs a genome-wide scan looking for SNPs that are significantly associated with the trait of interest while adjusting for covariates such as sex, age, and genetic principal components. Due to the large number of tests in GWAS, the convention is to use a stringent *p*-value threshold of 5 × 10^−8^ to account for multiple testing correction. Different from sequencing-based studies, a GWAS typically has a larger sample size due to the lower cost of microarray genotyping, but it is better powered to examine common variant associations than those for variants with lower frequencies due to poor imputation quality of rare variants, and a lack of ability for common variants to tag rare variants through LD.

Despite the simplicity, GWAS have identified tens of thousands of associations for numerous diseases and traits [79]. In particular, the recent emergence of large population-based biobanks (e.g., UK Biobank [1]) with comprehensive genotype and phenotype data, coupled with meta-analysis techniques [80] that allow a combination of summary-level association results across multiple independent cohorts, provides a golden opportunity for human geneticists to investigate the genetic basis of many human traits. It has been shown that GWAS-informed genes for disease traits are more likely to be drug targets [81]. Polygenic risk scores (PRS) based on large GWAS have shown substantially improved prediction accuracy and may have great potential for applications in the clinical setting [82].

GWAS also has some inherent limitations. One major challenge in population-based GWAS is the unadjusted confounding due to population stratification where different ancestry groups differ in both variant allele frequencies and the trait under study. In addition, recent evidence suggests that parental genotypes can be a major confounder for genetic associations identified in GWAS [83]. A person’s genetic variants exist in both himself/herself and the biological parents. Thus, these variants can affect a person’s phenotype both directly (through the inherited genetic variants) and indirectly (through the parents and the environment they create). GWAS results from a population cohort are a mixture of both the direct and indirect effects [84]. Because of these limitations, family-based GWAS, which investigate genotype–phenotype associations within families (e.g., between siblings), have gained renewed popularity [85]. Within-family GWAS is more robust to population stratification compared to studies conducted on unrelated individuals. Leveraging family data with shared environment also improves estimation of direct and indirect genetic effects, which provides more complete insights into the genetic basis of human complex traits [85,86]. However, statistical power remains moderate in family-based GWAS due to the limited number of families even in large biobanks.

Since the proportion of complex trait variance explained by the additive genetic components in GWAS is often smaller than heritability estimated from twin studies, gene–gene interactions have been hypothesized to partially account for this discrepancy [87,88]. However, testing all pairwise (or higher order) SNP interactions is computationally challenging and will severely reduce statistical power. Additionally, recent studies suggested very limited evidence for common SNP epistasis in complex trait genetics [89,90]. However, a growing literature suggests that both common and rare variants contribute to the risk of many diseases, and there may be a polygenic background for even rare “Mendelian-type” diseases [91,92]. For example, numerous genes harboring rare pathogenic variants as well as intergenic regulatory SNPs with higher frequencies have been implicated in diseases such as congenital heart disease and ASD [27,93,94,95,96,97]. It remains an open question whether the common, potentially polygenic genetic background can explain the incomplete penetrance of rare causal variants [98,99]. Increasing samples of WGS data in population biobanks (e.g., UK Biobank and All of Us) as well as ascertained disease cohorts (e.g., Simons Simplex Collection) will provide new opportunities for studying how common and rare variants jointly shape complex human phenotypes [100].

## 7. Disease Risk Prediction

A key goal in human genetic research is to identify individuals at higher disease risks for early screening and intervention. Thanks to the widely accessible summary-level data from GWAS, PRS models that can be trained directly using GWAS summary statistics have quickly gained popularity in recent years. In a nutshell, a PRS is a weighted (by variant effect sizes) sum of risk allele counts across a (possibly large) number of SNPs. It quantifies the genetic predisposition of disease risk for an individual and thus can be used to stratify individuals into high and low risk groups [82].

Methodological challenges in computing PRS reside in estimating the highly polygenic yet typically weak SNP effects for most complex traits and accounting for extensive LD in the human genome. Recently, penalized regression models that re-estimate SNP effects from GWAS summary statistics while explicitly modeling LD have been shown to effectively improve the predictive performance of PRS [101,102,103], and novel resampling approaches now allow model fine-tuning without individual-level genotype and phenotype data [104]. Additionally, Khera et al. convincingly demonstrated that individuals with very high PRS show substantially elevated coronary artery disease risk that is comparable to having monogenic mutations with large effects [105]. These studies showcase a promising future for PRS application in disease prevention and early intervention.

However, challenges remain before clinical use of PRS becomes a reality. Currently, the vast majority of published GWAS have been conducted on the non-Hispanic white population [106]. PRS trained from European samples are known to have drastically reduced prediction accuracy in non-European populations [107]. In addition, substantially reduced predictive performance has been observed across different demographic groups even within an ancestry population [108]. Similar reduction of PRS predictive power is also observed within families (e.g., between siblings), suggesting that a substantial fraction of genetic association estimated from GWAS may be mediated by the family environments [84]. To better understand the biological mechanisms of genetic associations underlying the trait-associated loci, it will be critical to distinguish causal effects from environmental (and familial) confounding, and to explain the lack of portability of PRS between the sexes, across the social economic status spectrum, and in diverse ancestral populations before we can appropriately apply PRS to the general populations.

## 8. Gene Therapy

A primary objective of human genetic studies is to uncover novel genetic etiology to disease and elucidate pathomechanistic features to develop meaningful therapies for patients. Among the most-promulgated forms of novel therapies stemming from human genetic studies is gene therapy, which seeks to alter the biological properties of living cells by modifying or modulating the gene function and expression in cells [109]. Being potentially curative, gene therapy has the capacity to spare patients’ years of drug intake in favor of one-time treatments with lifelong efficacy.

While gene therapy techniques can target both somatic and germline cells, ethical concerns about introducing heritable changes to humans have prevented the U.S. Food and Drug Administration (FDA) from approving any therapies targeting germline cells. Different strategies for different types of diseases have been developed in past decades: (a) inserting a functional copy of a gene to restore the biological function disrupted by a deficient copy [110]; (b) providing an interference molecular segment (i.e., small interfering RNA, suppressor gene, etc.) to inhibit the deficient gene function [111]; (c) correcting the deficient copy of a gene using genome editing techniques; and (d) adoptively transferring genetically engineered cells (e.g., hematopoietic stem cells or T cells) to restore or eliminate the dysfunctional cells [112].

Generally, drug development is divided into five steps: discovery, preclinical research, clinical research, FDA review, and post-market monitoring. This process is lengthy and expensive, taking up to 12–15 years with costs of more than USD 1 billion and increasing every year. At the same time, conventional drug development has slowed exponentially, with the number of new drugs brought to market per billion USD spent on research and development decreasing ten-fold since 1980 and fifty-fold since 1960 [113]. Thus, robust human genetic studies and integrative multi-omics analyses have become an attractive high-throughput, hypothesis-free methodology to identify potential targets and explicate pathomechanisms to better inform drug development [114]. Moreover, these targets feed into gene therapy development, which, with further study, may present a safe and adaptable system to provide curative therapies for a variety of genetic disorders. Currently, thousands of clinical trials for gene therapy targeting different diseases are ongoing in the US, but the gene therapy technologies are still in a constant state of development and improvement.

In a poignant example of this ‘base pairs-to-bedside’ approach to drug development, until 2017 sickle cell disease (SCD), one of the most common inherited blood disorders, had seen no therapeutic innovation to meet unmet clinical needs in over 20 years. Thanks to the progress of disease association analysis and advanced genetic engineering, more-specific drugs (i.e., Oxbryta and Adakveo) have become available in the past 3 years [115,116,117]. Since the SCD phenotype arises from a monogenic defect affecting the β-globin gene [118], the current strategies for gene therapy treatment are relatively straightforward. The defective β-globin gene function is corrected either by providing a fully functional copy of the gene or by restoring the expression of the γ-globin gene, a transitory paralog of β-globin appearing in fetal development. The approach for SCD requires gene modification in hematopoietic stem cells from the patient followed by transplantation of the functional cells. An ongoing clinical trial (ClinicalTrials.gov numbers, NCT03282656) showed a promising outcome, whereby the patient had prompt hematopoietic reconstitution after treatment [119]. There are many other inherited diseases with FDA-approved gene therapy treatments, including β-thalassemia [120], amyotrophic lateral sclerosis [121], autosomal dominant non-syndromic hearing loss [122], hemophilia A and B [123,124], retinal dystrophy [125,126,127,128,129], spinal muscular atrophy [130], and cystic fibrosis [131] (Table 2). With many more gene therapy treatments still in ongoing development or clinical trials, it is reasonable to expect significant growth in gene therapy applications as the technology matures and analytical genomic science further increases successful therapeutic yield.

## 9. Conclusions

The past decade has been the most fascinating era in the field of human genetics. We have witnessed unprecedented advances in biotechnologies for high-throughput omics, the creation of numerous global biobank cohorts with rich genotypic and phenotypic information, and the emergence of sophisticated statistical and computational methods for disease gene mapping and risk prediction. In this review, we introduced the state-of-art methods for research applications based on the study design (i.e., population, or trio-based family), genomic technology (i.e., WES, WGS, and GWAS), and the type of genetic variations under investigation (i.e., de novo, recessive, transmitted, X-linked, and digenic). We also discussed the current best practices of genomic study in human disorders—gene therapy—and summarized currently available treatments for diseases (Table 2).

As demonstrated in many studies, genetic variations alter patient responses to clinical treatments [142,143,144]. Although much progress has been made in identifying the genetic etiologies of many complex diseases, additional investigation is required to functionally connect most genetic variants with disease phenotypes through molecular pathomechanisms. The advent of GWAS/WES and, more recently, WGS has equipped molecular geneticists with the tools needed to decipher the genetic etiologies of rare and complex diseases. Current multi-omics studies using single-cell RNA-sequencing, ChIP-seq, and ATAC-seq have revealed more comprehensive complex biological molecules involved in the structure, function, and dynamics of a cell, tissue, or organism (reviewed in Ref. [145]). The integration of these novel technologies presents new hope in explicating the functional impact of many disease risk variants and the genetic pathology of complex disease traits. For many patients, this represents the end of a lifelong diagnostic odyssey preventing them from receiving precision therapy, understanding their prognosis, and making important life-planning decisions.

Many in the field speculate that, as WES/WGS becomes increasingly more common and affordable, increased understanding of variant–phenotype relationships and novel integrative genomic and pharmacogenomic therapeutic approaches tailored to patient-specific genetic information may revolutionize clinical care by increasing treatment specificity [146,147]. Quantitative phenomics is a critical component of the evolving integrative genomic approach. Standardized human phenotype annotation databases [148,149] and novel phenotype clustering algorithms [150,151] are developing to enable much more comprehensive and intelligent phenomics analysis. Transitioning to high quality, electronic, and increasingly standardized phenomics information can improve the phenotypic characterization of various heterogeneous disorders and identify associations between certain genetic variants and their respective clinical outcomes or presentation. This thereby provides better prognostication and clinical management, particularly of disorders with highly varied and poorly differentiated intra-disorder phenotypes [152,153]. Incorporating patient genetic information into clinician-friendly data platforms (i.e., electronic medical records) will maximize drug efficacy and minimize adverse effects, enriching precision medicine in practice [154]. The interface between genomic information and electronic health records coupled with increasingly improved methods can facilitate more precise discovery of genetic variants to guide more accurate therapeutic decisions in the future.

## Figures and Tables

**Table 2:** Commercially Available Gene Therapies in the U.S. in Alphabetical Order (2021) [132].

Name	Manufacturer	Target Disease	Gene of Interest	FDA Approval Date
Abecma (*idecabtagene vicleucel*)	Celgene Corporation (Bristol-Myers Squibb Company)	Relapsed or refractory multiple myeloma	BCMA (B-cell maturation antigen)	March 2021 [133]
Breyanzi (*lisocabtagene* *maraleucel*)	Juno Therapeutics (Bristol-Myers Squibb Company)	Relapsed or refractory large B-cell lymphoma	CD137 (4-1BB TNF- receptor) and CD3-zeta	February 2021 [134]
Imlygic (*talimogene laherparepvec*)	BioVex (Subsidiary of Amgen)	Melanoma (unresectable cutaneous, subcutaneous, and nodal lesions)	GM-CSF (immune stimulatory protein)	October 2015 [135]
Kymriah (*tisagenlecleucel*)	Novartis Pharmaceuticals Corporation	Pediatric B-cell precursor acute lymphoblastic leukemia (ALL)	CD137 (4-1BB TNF- receptor) and CD3-zeta	August 2017 [136]
		Relapsed or refractory large B-cell lymphoma in adult	CD137 (4-1BB TNF- receptor) and CD3-zeta	May 2018 [136]
Luxturna (*voretigene* *neparvovec-rzyl*)	Spark Therapeutics	Retinal dystrophy (biallelic RPE65 mutation- associated)	RPE65 (human retinal pigment epithelial 65 kDa protein)	December 2017 [137]
Provenge (*sipuleucel-t*)	Dendreon Corporation	Asymptomatic or minimally symptomatic metastatic castration-resistant prostate cancer (mCRPC)	ACP3 (prostate acid phosphatase)	April 2010 [138]
Tecartus (*brexucabtagene* *autoleucel*)	Kite Pharma	Relapsed or refractory mantle cell lymphoma (MCL) in adult	CD28 and CD3-zeta	July 2020 [139]
		Relapsed or refractory B-cell precursor acute lymphoblastic leukemia (ALL) in adult	CD28 and CD3-zeta	October 2021 [139]
Yescarta (*axicabtagene* *ciloleucel*)	Kite Pharma	Relapsed or refractory large B-cell lymphoma	CD28 and CD3-zeta	October 2017 [140]
		Relapsed or refractory follicular lymphoma	CD28 and CD3-zeta	March 2021 [140]
Zolgensma (*onasemnogene abeparvovec-xioi*)	Novartis Gene Therapies (Formerly AveXis)	Spinal muscular atrophy (Type I)	SMN1 (human survival motor neuron 1 protein)	May 2019 [141]

Licensed gene therapies in the U.S. approved by the Office of Tissues and Advanced Therapies (OTAT) as of 26 October 2021. Name = trade name (proper name); Manufacturer = name of pharmaceutical / biotechnology company licensed; Target Disease = FDA approved indication(s) excluding disease state(s) in ongoing clinical trials; Gene of Interest = biological/therapy target (and encoded protein if applicable); FDA approval date = indication license date based on FDA approval letters.

## Data Availability

Not applicable.
